# Functional Disorders of Digestion1Read before the Medical Society of the County of Cattaraugus, at Olean, N. Y., February 5, 1901.

**Published:** 1901-04

**Authors:** Clarence King

**Affiliations:** Machias, N. Y., Physician to the Cattaraugus County Hospital


### FUNCTIONAL DISORDERS OF DIGESTION.[1]

By CLARENCE KING, M. D., Machias, N. Y.,
Physician to the Cattaraugus County Hospital.

THE three great organs of digestion are the small intestines, the liver and the pancreas; deprived of either one of these, or with the function of either seriously and continuously interfered with, the person soon dies. The auxillary organs of digestion are the stomach and the salivary glands of the mouth. This statement is greatly at variance with our former ideas of digestion, for it has been thought until recently that the stomach was the organ principally concerned. But that such is not the case recent observations amply demonstrate. It is now generally acknowledged that by far the greater part of digestion takes place in the intestines, some authorities placing it as high as 90 per cent. No class of food stuff depends entirely upon the stomach for its digestion, but all may be acted upon further along in the alimentary passage. For this reason, we must admit, the stomach resembles the mouth with its unimportant glandular appendages, more than it does the great intestinal laboratory or factory with its very essential secretions, and great digestive activity. But it differs from both in being the storehouse which receives the food and slowly filters it into the intestines in such quantities as to meet the possibilities of the intestinal fluids for digestion. The pylorus guards the entrance to the intestine and regulates the supply of raw material to this digestive workshop. Without this safeguard the intestine would be flushed with fluid or its upper part at least overloaded with semi-solid material after each meal, before the necessary digestive fluids could be secreted in sufficient quantity to effect digestion.

The function of the stomach aside from that mentioned, is mainly to supplement the teeth in finely dividing and macerating the food so it can be brought more intimately in contact with the digestive fluids. In this respect it resembles somewhat the gizzard of the fowl. But it has a digestive function as well, the same as the bucal cavity, although as stated, this is not necessary to life. That the stomach is not essential to digestion is proven by Schlatter's patient who suffered complete excision of the stomach with little ill effect. Its digestive ability is therefore limited, perhaps its most important function being to dissolve the fibrous connective tissue of meats, thus freeing the muscular fibers and fats for intestinal digestion. It also coagulates casein and dissolves albumin, but upon starch, sugar and fats, it has no influence.

1. Read before the Medical Society of the County of Cattaraugus, at Olean, N. Y., February 5, 1901.

The saliva of the mouth converts starch into glucose and this function of saliva remains active for a time in the stomach, although acids soon stop it. In the same way the acid and ferments of the stomach continue their activity in the upper intestine, until they are finally neutralised and rendered inert by the alkaline intestinal and pancreatic juices. The secretion of the intestinal glands converts starch into sugar; as also does the pancreatic secretion; and it is probably the agent principally concerned in the digestion of meats and albumin. The best known function of the pancreatic juice is its action in emulsifying fats; but this is by no means its only function in digestion. It is now positively known that it has an important action upon the digestion of the nitrogenous principles and that the albuminoids, casein, gluten and muscular fibers are all digested by it. The action of the bile in digestion is not as well known as the other fluids, but that it has an important function there can be no doubt. It probably assists in the digestion of all the various articles of food or is essential to their absorption and assimilation. Intestinal putrefaction is prevented by it and peristalsis regulated. But aside from these facts there is little positively known about the influence of either the bile or pancreatic fluid.

The symptoms which usually accompany disturbances of digestion and to which the term dyspepsia is generally applied are: pain or distress at the epigastrium, a sensation of weight or fulness in the stomach after eating, impaired appetite, belching of gas, a furred tongue and constipation. With these there may be vomiting, pyrosis, abdominal tympanites, vertigo and other abnormal sensations. These symptoms may be present in certain organic diseases of the stomach or intestines and may for a time be the only evidence of disease; they are also common to several dissimilar functional disorders. If due to organic disease, there will generally be found some other distinctive symptom by which the true nature of the complaint can be recognised; but when functional, they often form our only guide to diagnosis. And too often even at the present time an attempt at an exact diagnosis is not made, but the symptoms are accounted for by the generic term "dyspepsia," or a "little stomach trouble." These are the cases which were formally treated in a routine manner with hydrochloric acid, pepsin, nux vomica, gentian and alkalies, sometimes with and sometimes without benefit.

Recent investigation has succeeded in clearing up much of the uncertainty which surrounded the diseases of the stomach. But while the diseases of the stomach and stomach digestion are comparatively well known, the diseases or disturbances of digestion below the pylorus are very poorly understood. This is no doubt due to the fact that no way has been devised by which we can obtain for examination the contents of the bowel at different parts and during different stages of the digestive process. Our knowledge of intestinal digestion is mostly derived from observations upon the lower animals and our ignorance concerning it, as it occurs in the human being or its condition in any individual patient, whether normal or disturbed, is too apparent for any one to question. Doubtless the derangements of intestinal digestion, pure and simple, are not as frequent as those of the stomach, for the reason that the intestine is better protected from severe and improper usage than the stomach. All food which enters the intestine has first to pass through the stomach and its physical and chemical characters are there somewhat changed and corrected, to make it suitable for the intestine. Thus the extremes of temperature are corrected, and hot or cold fluids or foods are brought to the happy medium of blood heat by the stomach, and are passed to the intestine at the temperature most favorable for digestion and least likely to cause harm. Strong irritants are retained in the stomach until they are largely diluted and rendered harmless; and indigestible substances are made less dangerous by having their rough edges wore off and their surfaces coated with mucus before they enter the intestine. This careful protection of the really important part of the digestive tract is in accordance with the wise provision of nature, which throws extra safeguards around the vital processes of our being in order to protect them from serious injury.

Stomach digestion begins immediately after the ingestion of food and is completed normally in from two to three hours, according to the character of the food. It is an intermittent function and regulated by the frequency of the meals. But intestinal digestion is practically a continuous process for it is both slower and much more perfectly performed. The secretions may be thrown out at intervals, but the solvent or digestive action continues so long as there is anything within the digestive area, and this is continued during sleep the same as during the wakeful hours. Advantage may be taken of this fact in treating certain cases of insomnia, since a full meal at bedtime of some bland and unirritating articles of diet will often produce sleep much better than opiates; while indigestible food will have the opposite effect.

The majority of cases of gastric indigestion are those of overstimulation; hence stimulants like nux vomica and ginger can do no good. For these sedatives are required, of which bismuth is the type, and, as over-stimulation produces an excess of acid, it is necessary to combine an alkali, as magnesia, with the sedative. But on the other hand, if the urine contains indican without obvious extra-intestinal cause, as will be explained later, we should institute treatment by measures intended to stimulate the gastric and biliary secretions. For this purpose the bitter tonics and chologogues may be tried, or the mineral acids, or Prof. Porter's favorite prescription, which contains ox-gall, pancreatin, caffeine, compound extract colocynth and strychnia. If improvement does not occur from these measures a more thorough examination should be made by the passage of the stomach tube and an analysis of the stomach contents.

The anatomical diseases of the stomach are five in number, and it will be necessary to consider and exclude them in order to reach a diagnosis of a purely functional disorder, for they give rise to digestive disturbances the same as the functional diseases. They can generally be diagnosticated by their physical signs without resource to the stomach tube. Ulcer and cancer we will dismiss without further notice, although chemical analysis is an aid to diagnosis in the early stages. Displacements and enlargements of the stomach must be made out by palpation and percussion, either with or without inflation of the stomach with gas. Obstruction at the orifices is diagnosticated by palpation in conjunction with the subjective symptoms, and the use of the esophageal sound when it is the cardiac orifice which is obstructed. Of gastritis, I will speak presently.

The motor function of the stomach is frequently disturbed, especially as a complication of other diseases. Atony may be detected by the splashing sound produced by short, quick blows with the finger over the stomach at a time when the organ normally is empty. Of course, it indicates fluid in the stomach and generally dilatation also. It gives rise to dyspeptic symptoms, minus the pain, and obviously is not benefited by pepsin, acid or alkalies, but by general tonics, lavage, electricity and massage. Eructation, pyrosis, regurgitation and rumination are perversions of the motor function, the first two of which are common symptoms of other diseases and are generally helped by treatment of the primary disease; the others are rare. Spasms of the orifices are also rare and can scarcely be distinguished from organic strictures; their treatment is mostly surgical, although bromides and opiates have been thought to have some beneficial effect.

To further investigate the diseases of the stomach, we need the stomach contents taken at a certain time after a specified meal, at which time we know the normal condition of digestion. The stomach contents are obtained for examination by passing the tube one hour after the Boas-Ewald test breakfast, which consists of one or two rolls, or a slice of bread and a cup of tea or a glass of water. After the tube is introduced the patient should make compression of the abdomen and express as much of the stomach contents as possible. If difficulty is experienced in getting the contents, the tube may be shaken violently or a suction effect employed by means of a rubber bulb. Care should be taken to have the head well thrown back to avoid a sharp bend in the tube in the pharynx.

The stomach contents thus obtained should be measured, but as it is impossible to be sure that all of the contents have been obtained, the following method has been devised to estimate the amount remaining. After obtaining all that is possible with the tube two hundred cubic centimeters of clear water is poured into the stomach and after a few minutes withdrawn. The acidity of the contents expressed and of this stomach, washing is determined separately by the method I will soon describe and the amount of contents remaining after expression is figured from these results, which added to the measured amount of contents expressed gives the total contents. Normally, this should be about 125 to 150 cubic centimeters.

To obtain the amount of the contents remaining after expression, we should multiply the acidity of the diluted contents by 200, (the amount of water introduced,) and divide the product by the difference between the total acidity and the acidity of the diluted contents. If the total contents is too high, it shows the motor function is at fault and that some degree of atony is present, but if it is too low there will generally be found hyperchlohydria and increased peristaltic activity. The motor function can also be tested without the use of the tube in the following manner: 15 grains of salol in capsules are given soon after eating and the urine examined at intervals for salicyluric acid. This is easily detected by moistening filter or blotting paper with the urine and treating with a drop of a 10 per cent. ferric chloride solution. If salicyluric acid is present there will be formed a violet color around the drop of iron solution.

The stomach contents obtained by expression should first be examined for mucus. That which floats on top should be removed, for that is from the throat or esophagus and has nothing to do with the stomach. Then with a fine wire or a splinter of wood, the expressed material should be searched for shreads of mucus, which may be intimately mixed with it. If any mucus is found it shows inflammation of the gastric mucosa or gastritis in some form and the case is therefore out of our present consideration.

The expressed contents we have obtained should then be filtered and the filtrate examined for acids. Congo red test paper is changed to dark blue if free acid is present and dimethyl-amido-azo-benzol paper is turned red if that free acid is hydrochloric. If hydrochloric acid is present the amount should be estimated, which is done by adding a few drops of a 1 per cent. solution of dimethyl-amido-azo-benzol to five cubic centimeters of the filtrate, and titrating with enough decinormal solution of sodium hydrate to effect discolorisation. The amount in cubic centimeters of sodium hydrate solution used multiplied by 20, will represent the amount of free hydrochloric acid to 100 cubic centimeters of filtrate. Normally this should be between 10 and 20. To obtain the total acidity a few drops of a 1 per cent. solution of phenol-phthalein should be added and the titration with sodium hydrate continued until a slightly red color appears. The total amount of the sodium hydrate solution used multiplied by 20, gives the total acidity; in health this is between 50 and 60. If these figures are increased hyperchlorhydria is present. This is generally due to simple nervous or mental causes or to errors in diet, but may be caused by ulcer. It is therefore of value in the differential diagnosis of ulcer from cancer, for with cancer hydrochloric acid is diminished or absent. When hyperchlorhydria is functional its treatment consists in the administration of nerve sedatives, regulation of the diet, lavage and the use of alkalies at the end of stomach digestion, to neutralise the excess of acid. If mental overwork, grief or worry is a factor in the case, it should be removed if possible and a month's vacation under change of conditions taken.

Deficient secretion of hydrochloric acid and lessened acidity may be due to cancer, acute or chronic gastritis, dilatation, atony or atrophy of the stomach, neurasthenia and hysterical or atonic dyspepsia, so-called. If the test papers show a free acid present, but no hydrochloric acid, then the acid is organic and may be butyric, acetic or lactic. Butyric acid is recognised by its strong odor of rancid butter, acetic acid also by its odor, and lactic acid by the following method: 5 cubic centimeters of the filtrate are diluted with 50 cubic centimeters of water, and one or two drops of a 5 per cent. solution of sesquichloride of iron added. If lactic acid is present there will be a distinct greenish-yellow color obtained. The significance of these acids is that they point to the probable presence of carcinoma, but certain precautions must be taken. Thus, as regards acetic acid, we must exclude the previous ingestion of alcoholic drinks; and as regards the other two must have washed the stomach thoroughly and given a test meal, preferably of soup, before passing the tube.

The normal acidity of the gastric juice is due to three factors—namely, free hydrochloric acid, loosely combined hydrochloric acid and certain acid salts; in diseased conditions there may be organic acids present also. The ferments require for their activity hydrochloric acid, either free or combined. Free acid does not appear until after a certain amount has combined with the albuminous food; and, hence, if free acid is absent, it is necessary to determine if there is any combined acid in order to know if peptic activity is possible. If there is any hydrochloric acid at all secreted pepsin, is secreted also and is active to the extent of the acid. This rule is almost absolute. Consequently, we need not concern ourselves much with the pepsin. But the combined acid may be determined by finding the amount of free acid and acid salts and subtracting from the total acidity. The free acids and [illegible]id salts together, are found by adding two or three drops of a one-per cent. solution of alizarin to 5 cubic centimeters of ston[illegible] filtrate, and enough of the decinormal sodium hydrate solution to obtain a clear violet color. The amount of sodium hydrate solution used, multiplied by 20 gives the acidity of the free acids and acid salts together. Obviously, the absence of combined hydrochloric acid has no great diagnostic value, for it can be absent only in those diseases where there is no free hydrochloric acid and only occasionally is wanting in them. But it has an important bearing on prognosis and on the dietetic indications, for if all hydrochloric acid and consequently pepsin and its zymogen is absent or inactive it shows that glandular activity is totally and permanently destroyed, and that such food must only be ingested as will quickly come to pancreatic digestion and require no preliminary preparation in the stomach. The examination for pepsin, starch, sugar, bile and blood is seldom required, and will not be here described.

Intestinal indigestion, as a functional disorder, is perhaps most frequent in persons past middle age and in those persons whose general health is below the normal standard, as the result of previous sickness, anemia, neurasthenia, syphilis or tuberculosis. But it is also frequent in infants and children when it is often overlooked and the symptoms attributed to other causes. In infants the shape and relative position of the stomach are such as to favor the early discharge of its contents into the intestine; and, as it is deficient in acid and ferments, it is unable to properly prepare solid food for intestinal digestion, and putrefaction and indigestion results. In men the influence of alcohol and tobacco in causing indigestion, both gastric and intestinal, can not be doubted; while in women constipation is perhaps its most potent cause. Of course, any disease which interferes with the proper secretion of the intestinal fluids which are concerned in digestion will give rise to indigestion. Errors of the portal circulation caused by cancer or cirrhosis, chronic hepatitis or catarrh of the biliary ducts may also produce it. Diseases of the pancreas of whatever nature are attended by intestinal indigestion, and chronic catarrh of the small intestines, by interfering with the secretion of the intestinal glands, may give rise to it. Many other causes have been assigned, but they are far-fetched and rather indefinite.

The subjective symptoms of intestinal indigestion are similar to those produced by disturbances of the gastric function and, indeed, the two are often, if not generally, associated. Either may be acute or chronic. When acute, there is generally pain, vomiting, diarrhea and fever, and in infants these symptoms are sometimes quite severe. Convulsions may be initial symptoms, or they may occur after a few hours and be repeated several times. In older children and adults, in addition to the pain and vomiting, there may be tympanites, a furred tongue and a history of the ingestion of some food or substance difficult of digestion. The hot weather of summer is an important factor in producing gastro-intestinal indigestion, especially in infants who are bottle fed, due in part to some fermentation change in the milk, and in part to the effect of the heat itself in destroying or enfeebling the equilibrium of the nervous centers.

Chronic indigestion is much more frequent in adults than the acute form, although in infants and children the reverse is undoubtedly true. At first there is a feeling of weight or distress in the stomach after dinner or after eating certain articles of food, and an uncomfortable fulness in the abdomen is complained of. There is frequently a coated tongue and belching of gas, and constipation is a very common feature. So insidiously do the symptoms develop, it is impossible to assign a time as the beginning of the trouble, but they increase in severity and may last indefinitely. When the patient finally presents himself for treatment, there is generally a poor appetite, with more or less pain after eating, and, perhaps, headache, vertigo, palpation of the heart, disturbed sleep and pyrosis. But sour eructations or the raising of sour fluid from the stomach has only a general symptomatic value and does not necessarily mean an excess of acid. There is seldom any great loss of flesh in these diseases unless there is some other serious disease to which indigestion is secondary, or the disease has lasted a long time and been unusually severe.

We have already intimated that gastric and intestinal indigestion are frequently, if not usually, associated, but it is often possible to determine which predominates. With gastric indigestion the pain and tenderness is referred to the epigastrium or the region of the stomach, while with intestinal indigestion it is about the umbilicus. With the former there is either diarrhea or constipation, or the two alternate; but with the latter constipation is nearly always present, and often the feces are passed as hard balls, due to an associated colitis. Headache, vertigo and palpitation are, perhaps, more common with intestinal disorder, and represent a toxemia from absorption of gases of putrefaction in the intestine. Other disturbances of the cerebrum, as insomnia, mental inertia, hypochrondia, perversions of the special senses and epileptiform convulsions, all point to intestinal indigestion as their origin; while sour eructations, pyrosis and frequent vomiting, are symptoms of gastric origin. Slight icterus, or the presence of bile or its coloring matter in the urine, shows that the function of the liver is interfered with, and the bile does not reach the intestine to aid in digestion. In like manner fat in the stools indicates disease of the pancreas, probably organic, but it may be possible there are functional disorders of this organ as well with which we are not acquainted. And finally, if the urine deposits a large amount of urates upon cooling, or if the extremities or body has the sensation of being swelled or bloated from capillary engorgement when no dropsy is present, then we may know the disease is intestinal rather than gastric.

I have said that we are unable to analyse the intestinal contents for defects in intestinal digestion, but we, nevertheless, have a test which gives us positive information in regard to this part of the intestinal tract. If putrefaction, and consequently indigestion, is present in the intestinal canal, indican appears in the urine in abnormal quantity and can readily be detected by analysis. The hydrochloric acid of the gastric juice is the normal intestinal antiseptic, and it is in those cases where this acid is deficient that putrefaction is most certain to occur and indican be present in the urine. But it most frequently occurs in those diseases of the small intestine, which are attended by diminished peristalsis from a lessened amount of bile, but is never found in disease of the large intestine alone. And, lastly, indican may attend albuminous putrefaction in other places than the intestine, as in empyema, putrid bronchitis, gangrene, and occasionally in abscess. Hence, it will be seen that the examination of the urine for indican is very important, and that it has a clinical significance barely second to albumin or sugar. If we are able to exclude this last source in any individual case, its value as regards indigestion is plain. If tests are made indican will be found oftener than either sugar or albumin, for the conditions which produce it are common. There are a number of tests for indican which may be employed, but the following is as satisfactory as any: About a drachm of urine is poured in a test tube and an equal amount of strong hydrochloric acid added; to this two or three drops of a 1 per cent. solution of permanganate of potash are added, when a blue ring of indigo is found. If chloroform is added, this will be carried to the bottom of the tube and the chloroform colored blue as the indigo is soluble in chloroform.

The proper dietetic restrictions in the treatment of functional indigestion are of the greatest importance in securing success, but first the alimentary canal must be thoroughly emptied. For this purpose chologogues may be used, or one or two teaspoonfuls of glycerine in an equal amount of sweet oil every four hours until a satisfactory effect is produced. Then in way of food we should avoid that which is difficult to digest, or leaves a large residue to pass through the intestines and be subject to putrefaction. Normally, we require a mixed diet for perfect nutrition and the amount of the vegetable foods should predominate. But animal foods are always more easily digested than vegetable, and they also leave less residue as foreign matter. Hence, in difficult or disturbed digestion animal foods should be mostly used, while course and bulky vegetable foods should be avoided. But we should not go to the extreme of using animal foods alone, for the system requires some vegetable constituents for blood formation and without them anemia will result. This fact should be remembered in treating diabetes mellitus. Of animal foods beefsteak stands at the head; it is digested and utilised to the extent of about 98 per cent., according to one authority, while raw eggs are second, 97 per cent. and milk third at 96 per cent. On the other hand, oat meal, in diseased conditions, is practically indigestible and its laxative effect in health is largely due to the great proportion of waste matter. Besides, it is particularly prone to putrefaction if evacuation does not soon take place. Fruits are also bad, as a rule, for persons with feeble digestion, because they are apt to set up fermentation, with formation of toxins, which finally attack the proteids and thus destroy the most important elements of nutrition.

The presence of urates or uric acid in the urine in increased amount, denotes either increased retrograde tissue changes or the failure of complete oxidation of the proteids of the food. If the urates, without obvious structural cause in other parts, are in sufficient quantity to deposit upon cooling of the urine, then it is evident the amount of the nitrogenous elements ingested is in excess of the ability of the digestive apparatus and that that excess, as it passes through the portal circulation, is changed by the liver to urea. Hence, if due to digestive trouble the proper course would seem to be to cut down the amount of proteids ingested, and at the same time to stimulate intestinal digestive activity. As a rule, fats should also be avoided, for they are apt to increase the stomach trouble; and starch and sugar should be reduced as much as possible. But in many cases the experience of the patient as regards food is a safe guide to follow.

As regards drugs I have little to say at this time. Of course the bowels should be kept active by laxatives and of these each one has his favorite. Obviously, it is impossible to specify remedies in a general way for the indigestion, for the causes are various and their treatment dissimilar. But we can say that pepsin is falling into disuse and that pancreatin is taking its place. Caffeine or coffee is said to double the activity of pancreatin, but of this I think there is some doubt. Ox-gall is not used as much as it should be, and alkalies are not employed by many with a clear understanding of their indications. Acids and gastric sedatives have their uses and can not be supplanted; as, likewise, lavage and electricity. But the profession has not generally learned that many cases are pure neuroses and must have nerve sedatives and measures to build up the general health.

If physicians would use the same care and intelligence in dealing with the functional digestive disorders, as they do in other diseases, these cases would not stand as a reproach to the profession, and we would not so often hear the expression that "the doctor can do nothing for dyspepsia."

---

It is reported that the French minister of war has ordered lectures to be given at army posts on the dangers of drunkenness.

